# Editorial: Intelligent robots for agriculture -- Ag-robot development, navigation, and information perception

**DOI:** 10.3389/frobt.2025.1597912

**Published:** 2025-04-08

**Authors:** Sierra N. Young

**Affiliations:** Civil and Environmental Engineering, Utah State University, Logan, UT, United States

**Keywords:** agricultural robotics, autonomous navigation, perception, precision agriculture, artificial intelligence, AI

## Introduction

Agriculture is undergoing a paradigm shift driven by global challenges such as climate change, labor shortages, and the increasing demand for sustainable food production. In response, intelligent robotics are emerging as a transformative technology, enhancing agricultural efficiency, precision, and sustainability. This Research Topic, “Intelligent Robotics in Agriculture -- Ag-Robot Development, Navigation, and Information Perception,” highlights advancements in agricultural robotics, including breakthroughs in system design, autonomous navigation, multi-sensor fusion, and machine learning applications. The collected works contribute significantly to the evolving landscape of smart farming by addressing critical challenges in agricultural automation.

## Advances in agricultural robotics

Robotic platforms tailored for agricultural environments require adaptability to diverse terrains, robustness in operation, and the ability to handle complex tasks. Zhang et al. present a bionic hexapod robot designed for agricultural field scouting, equipped with adaptive gait control for improved mobility across uneven terrains. Their study demonstrates the potential of legged robots in agricultural applications, showing enhanced stability and energy efficiency compared to traditional wheeled platforms.

Balabantaray et al. contribute to precision weed management by integrating deep learning with robotics. Their study introduces a YOLOv7-powered robotic system for targeted spraying of Palmer amaranth, significantly reducing herbicide usage while increasing accuracy in weed identification. The system demonstrates how AI-driven robotics can enhance environmental sustainability in modern agriculture.

## Autonomous navigation and sensor fusion

Autonomous navigation is a fundamental requirement for agricultural robots, enabling precise field operations. Mwitta and Rains explore the integration of GPS and visual navigation for Ackerman-steering mobile robots in cotton fields. Their research highlights the synergy between GPS-based global planning and deep learning-based local navigation using semantic segmentation, enhancing real-time adaptability in row-based crop navigation.

The combination of multiple sensor technologies, including LiDAR, RGB-D cameras, and GNSS, has been shown to improve the accuracy and robustness of robotic navigation in unstructured environments. Such multi-sensor fusion approaches allow agricultural robots to operate in dynamic field conditions with reduced reliance on human intervention. These advances represent a crucial step toward fully autonomous robotic farming systems capable of performing complex tasks such as seeding, fertilizing, and crop monitoring.

## Machine learning and imitation learning in agriculture

Artificial intelligence (AI) plays a pivotal role in advancing agricultural robotics, particularly in perception, decision-making, and control. Mahmoudi et al. provide a comprehensive survey on the role of imitation learning in agricultural robotics, demonstrating how robots can learn from human demonstrations to improve automation in complex agricultural tasks. Their work emphasizes the importance of machine learning in enabling robots to adapt to dynamic environments, enhancing their operational effectiveness in diverse agricultural settings.

Beyond imitation learning, AI-based models, including reinforcement learning and deep neural networks, are increasingly used to optimize robotic behaviors in agricultural environments. These technologies enable autonomous decision-making, allowing robots to adjust their actions based on real-time environmental feedback. The continued development of AI-driven agricultural robotics is expected to yield significant improvements in efficiency, scalability, and adaptability across various farming applications.

## Summary and outlook

By advancing robotic design, AI-driven decision-making, and autonomous navigation, these studies pave the way for a future where robotics plays a central role in ensuring global food security and sustainable farming practices. Future research should focus on improving the generalizability of robotic solutions across different agricultural domains, enhancing robot perception capabilities through multi-modal sensor fusion, and addressing cost-effectiveness for broader adoption. Moreover, ethical and regulatory considerations in AI-driven agriculture need further exploration to ensure responsible and sustainable deployment.

